# Genome sequence of the haloarchaeon *Haloterrigena jeotgali* type strain A29^T^ isolated from salt-fermented food

**DOI:** 10.1186/s40793-015-0047-4

**Published:** 2015-08-05

**Authors:** In-Tae Cha, Mi-Hwa Lee, Byung-Yong Kim, Yong-Joon Cho, Dae-Won Kim, Kyung June Yim, Hye Seon Song, Myung-Ji Seo, Jin-Kyu Rhee, Jong-Soon Choi, Hak-Jong Choi, Changmann Yoon, Seong Woon Roh, Young-Do Nam

**Affiliations:** Biological Disaster Analysis Team, Korea Basic Science Institute, Daejeon, 305-806 Republic of Korea; Division of Bioengineering, Incheon National University, Incheon, 406-772 Republic of Korea; Research Group of Gut Microbiome, Korea Food Research Institute, Sungnam, 463-746 Republic of Korea; ChunLab Inc., Seoul National University, Seoul, 151-742 Republic of Korea; Systems Biology Team, Center for Immunity and Pathology, Korea National Institute of Health, Cheongju, 361-951 Republic of Korea; Department of Food Science and Engineering, Ewha Womans University, Seoul, 120-750 South Korea; Graduate School of Analytical Science and Technology, Chungnam National University, Daejeon, 305-764 Republic of Korea; World Institute of Kimchi, Gwangju, 503-360 Republic of Korea; Korea University of Science and Technology, Daejeon, 305-350 Republic of Korea

**Keywords:** Haloarchaeon, *Haloterrigena jeotgali*, Genome sequence, Salt-fermented food, Jeotgal

## Abstract

*Haloterrigena jeotgali* is a halophilic archaeon within the family *Natrialbaceae* that was isolated from shrimp jeotgal, a traditional Korean salt-fermented food. A29^T^ is the type strain of *H. jeotgali*, and is a Gram-negative staining, non-motile, rod-shaped archaeon that grows in 10 %–30 % (w/v) NaCl. We present the annotated *H. jeotgali* A29^T^ genome sequence along with a summary of its features. The 4,131,621 bp genome with a GC content of 64.9 % comprises 4,215 protein-coding genes and 127 RNA genes. The sequence can provide useful information on genetic mechanisms that enable haloarchaea to endure a hypersaline environment.

## Introduction

An extremely halophilic archaeon, called a haloarchaeon, that is a member of the family *Natrialbaceae* [1] was isolated from various hypersaline environments such as soda and salt lakes, solar salterns, salt mines, salted soils, deep-sea brine, and various salt-fermented foods. Although high salinity is toxic to most cells, extreme halophiles are adapted to their hypersaline environments [2]. Most halophilic archaea require at least 1.5 M NaCl for growth and optimum growth occurs in the range of 3.1 to 3.4 M NaCl [3]. Since halophilic enzymes from the haloarchaea are generally considered to be active and stable at high salt concentrations, they have potential for biotechnological applications such as engineering for salt-resistant plants in agriculture, environmental bioremediation of organic pollutants and production of fermented foods. The genus *Haloterrigena* was first proposed by Ventosa et al. [4] with the reclassification of *Halococcus turkmenicus* as *Haloterrigena turkmenica* [4], and presently includes nine species: *H. turkmenica* [4], *H. thermotolerans* [5], *H. longa*, *H. limicola* [6], *H. saccharevitans* [7], *H. hispanica* [8], *H. jeotgali* [9], *H. salina* [10], and *H. daqingensis* [11], all of which are pleomorphic, Gram-negative staining, and red- or light pink-pigmented. However, the genus *Haloterrigena* is poorly characterized at the genome level.

A29^T^ (= KCTC 4020^T^ = DSM 18794^T^ = JCM 14585^T^ = CECT 7218^T^) is the type strain of *H. jeotgali* and was isolated from shrimp jeotgal, a traditional Korean salt-fermented food [9]. Although little is known about the roles of the haloarchaea during the fermentation process, the increasing genome information is expected to contribute to expansion of the understanding of their roles and halotolerant features. Here, we present a summary of the classification and features of *H. jeotgali* A29^T^ along with the annotated genome sequence.

## Organism information

### Classification and features

**Fig. 1:**
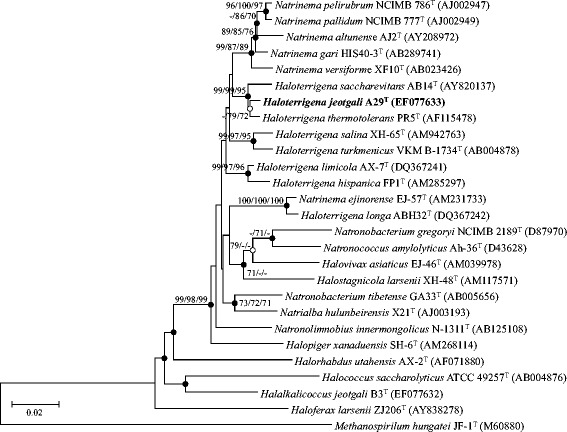
Phylogenetic tree based on the neighbor-joining (NJ) algorithm for the 16S rRNA gene sequences of strain A29^T^ and closely related taxa. Numbers at the nodes indicate bootstrap values calculated using NJ/minimum evolution (ME)/maximum likelihood (ML) probabilities. Filled and open circles represent nodes recovered by both ME and ML methods or by either method, respectively. *Methanospillum hangatei* JF-1^T^ served as an outgroup

A taxonomic analysis was conducted by comparing the *H. jeotgali* A29^T^ 16S rRNA gene sequence with the most recent release of the EzTaxon-e database [12]. Phylogenetic relationships between strain A29^T^ and closely related species were evaluated using MEGA6 program [13], and dendrograms were generated by the neighbor-joining [14], minimum evolution [15], and maximum likelihood [16] methods. A bootstrap analysis investigating the stability of the dendrogram was performed by obtaining a consensus tree based on 1,000 randomly generated trees. Strain A29^T^ showed the highest level of the 16S rRNA gene similarity to *H. thermotolerans* PR5^T^ (99.0 %), *H. saccharevitans* AB14^T^ (98.3 %), *H. limicola* AX-7^T^ (97.1 %), *H. turkmenica* 4k^T^ (96.8 %), *H. salina* XH-65^T^ (96.6 %), *H. hispanica* FP1^T^ (96.1 %), *H. longa* ABH32^T^ (94.9 %), and *H. daqingensis* JX313^T^ (94.6 %). The DNA-DNA relatedness between strain A29^T^ and the related strains *H. thermotolerans* PR5^T^, *H. saccharevitans* AB14^T^, and *H. limicola* AX-7^T^ was 23.2 %, 22.0 %, and 17.9 %, respectively. The 16S rRNA gene sequence similarity data and DNA–DNA relatedness value of less than 70 % [17] suggested that strain A29^T^ represents a distinct genospecies [9] (Table 1). The consensus phylogenetic tree based on the 16S rRNA gene sequences indicated that strain A29^T^ was clustered in a branch with other species of the genus *Haloterrigena* (Fig. 1).

**Table 1:** Classification and general features of *Haloterrigena jeotgali* A29^T^ [19]

MIGS ID	Property	Term	Evidence code^a^
	Classification	Domain *Archaea*	TAS [25]
		Phylum *Euryarchaeota*	TAS [26]
		Class *Halobacteria*	TAS [27, 28]
		Order *Natrialbales*	TAS [1]
		Family *Natrialbaceae*	TAS [1]
		Genus *Haloterrigena*	TAS [4]
		Species *Haloterrigena jeotgali*	TAS [9]
		(Type) strain A29^T^ (KCTC 4020, DSM 18794, JCM 14585, CECT 7218)	TAS [9]
	Gram stain	Negative	TAS [9]
	Cell shape	Rod	TAS [9]
	Motility	Non-motile	TAS [9]
	Sporulation	Not reported	
	Temperature range	17–50 °C	TAS [9]
	Optimum temperature	37–45 °C	TAS [9]
	pH range; Optimum	6.5–8.5; 7.0 − 7.5	TAS [9]
	Carbon source	Fructose, lactose, acetate	TAS [9]
MIGS-6	Habitat	Salt-fermented food	TAS [9]
MIGS-6.3	Salinity	35 % NaCl (w/v)	TAS [9]
MIGS-22	Oxygen requirement	Aerobic	TAS [9]
MIGS-15	Biotic relationship	Free-living	TAS [9]
MIGS-14	Pathogenicity	Not reported	
MIGS-4	Geographic location	South Korea	TAS [9]
MIGS-5	Sample collection	2006	NAS
MIGS-4.1	Latitude	Not reported	
MIGS-4.2	Longitude	Not reported	
MIGS-4.4	Altitude	Not reported	

^a^Evidence codes - TAS: Traceable Author Statement (i.e., a direct report exists in the literature); NAS: Non-traceable Author Statement (i.e., not directly observed for the living, isolated sample, but based on a generally accepted property for the species, or anecdotal evidence). These evidence codes are from the Gene Ontology project [29]

**Fig. 2:**
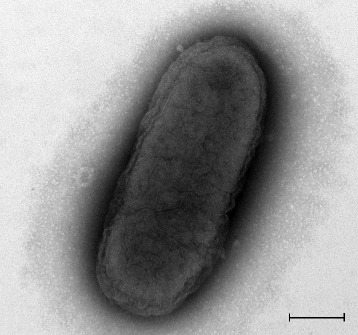
Transmission electron micrograph of *H. jeotgali* A29^T^. The scale bar represents 200 nm

*H. jeotgali* A29^T^ is Gram-negative staining, non-motile, rod-shaped (0.4 μm wide and 1.0 μm long) (Fig. 2), and grows in irregular clusters. Colonies cultured on complex agar medium were light red, circular, and measured 0.5 mm in diameter after 7 days at 37 °C. Growth occurred in the presence of 10–30 % (w/v) NaCl at temperatures ranging from 17–50 °C and in the pH range of 6.5–8.5. Optimal conditions for growth were; a NaCl concentration of 15–20 % (w/v), a temperature ranging from 37–45 °C, and a pH of 7.0–7.5. The isolate was catalase-positive and oxidase-negative and did not reduce nitrate to nitrite. Mg^2+^ was not required for growth. Cell lysis occurred in distilled water. This strain was able to hydrolyze casein and Tween 80 but not starch, gelatin, urea, or DNA. Anaerobic growth occurred in the presence of nitrate but not of sulfate, thiosulfate, dimethyl sulfoxide, or trimethylamine N-oxide. Fructose, lactose, and acetate—but not sucrose, glucose, citrate, or formate—were utilized as carbon and energy sources. Acid was not produced from fructose, lactose, acetate, sucrose, glucose, citrate, or formate. Strain A29^T^ was resistant to bacitracin, penicillin, ampicillin, chloramphenicol, and erythromycin, but was sensitive to novobiocin, anisomycin, and aphidicolin. The major polar lipids were phosphatidylglycerol, phosphatidylglycerol phosphate methyl ester, and mannose-2,6-disulfate(1–2)-glucose glycerol diether [9].

## Genome sequencing and annotation

### Genome project history

*H. jeotgali* strain A29^T^ genome was sequenced to obtain information regarding mechanism(s) or molecule(s) that confer adaption to a hypersaline environment and to identify the primary structure of potentially novel halophilic enzymes with relatively low similarity to those in the sequence database. The genome project and sequence were deposited in the Genomes OnLine Database [18] and GenBank (JDTG00000000), respectively. Sequencing and annotation were performed by ChunLab Inc. (Seoul, Korea). Project information and associated MIGS version 2.0 compliance levels [19] are shown in Table 2.

**Table 2:** Project information

MIGS ID	Property	Term
MIGS-31	Finishing quality	Improved-high-quality draft
MIGS-28	Libraries used	300-bp paired end (Illumina); 400-bp single end (Ion Torrent); 10 kb (PacBio RS)
MIGS-29	Sequencing platforms	Illumina MiSeq, Ion Torrent PGM, PacBio RS system
MIGS-31.2	Fold coverage	700.5×
MIGS-30	Assemblers	CLC Genomics Workbench 6.5.1, SMRT Analysis 2.1
MIGS-32	Gene calling method	GLIMMER 3.02
	Locus Tag	HL44
	GenBank ID	JDTG00000000
	GenBank Date of Release	June 20, 2014
	GOLD ID	Gi0069863
	BIOPROJECT	PRJNA236631
MIGS-13	Source material identifier	A29^T^
	Project relevance	Environmental and biotechnological

### Growth conditions and genomic DNA preparation

*H. jeotgali* A29^T^ was grown aerobically in DSM Medium 954 at 37°C. Genomic DNA was extracted and purified using a G-spin™ DNA extraction kit (iNtRON Biotechnology, Sungnam, Korea) according to the manufacturer’s instructions.

### Genome sequencing and assembly

The genome of *H. jeotgali* A29^T^ was sequenced from a total of 9,473,809 quality-trimmed sequencing reads (700.5-fold coverage) that combined 6,797,702 reads (473.8-fold coverage) from the Illumina MiSeq. 300 bp paired-end library (Illumina, San Diego, CA, USA); 2,617,102 reads (181.1-fold coverage) obtained using an Ion Torrent Personal Genome Machine (PGM) 318v2 chip (Life Technologies, Carlsbad, CA, USA); and 59,005 reads (45.7-fold coverage) from a PacBio RS 10 kb library (Pacific Biosciences, Menlo Park, CA, USA). Illumina and PGM data were assembled *de novo* with CLC Genomics Workbench 6.5.1 (CLC bio, Boston, MA, USA) and PacBio data were assembled with the HGAP2 algorithm in SMRT Analysis 2.1 (Pacific Biosciences). Resultant contigs were assembled with CodonCode Aligner 3.7 (CodonCode Corporation, Centerville, MA, USA). The final assembly yielded three scaffolds with 20 contigs spanning 4.1 Mb.

### Genome annotation

Open reading frames of the assembled genome were predicted using the Integrated Microbial Genomes-Expert Review platform as part of the Joint Genome Institute genome annotation pipeline [20]. Additional gene prediction and functional annotation were achieved using the Rapid Annotation using Subsystem Technology pipeline. Predicted ORFs were compared during gene annotation using NCBI Clusters of Orthologous Groups [21], Pfam [22], and EzTaxon-e [12] databases. rRNA and tRNA genes were identified using RNAmmer 1.2 [23] and tRNAscan-SE 1.23 [24] tools, respectively. Genomic features were visualized with CLgenomics 1.06 (ChunLab Inc.).

## Genome properties

**Fig. 3:**
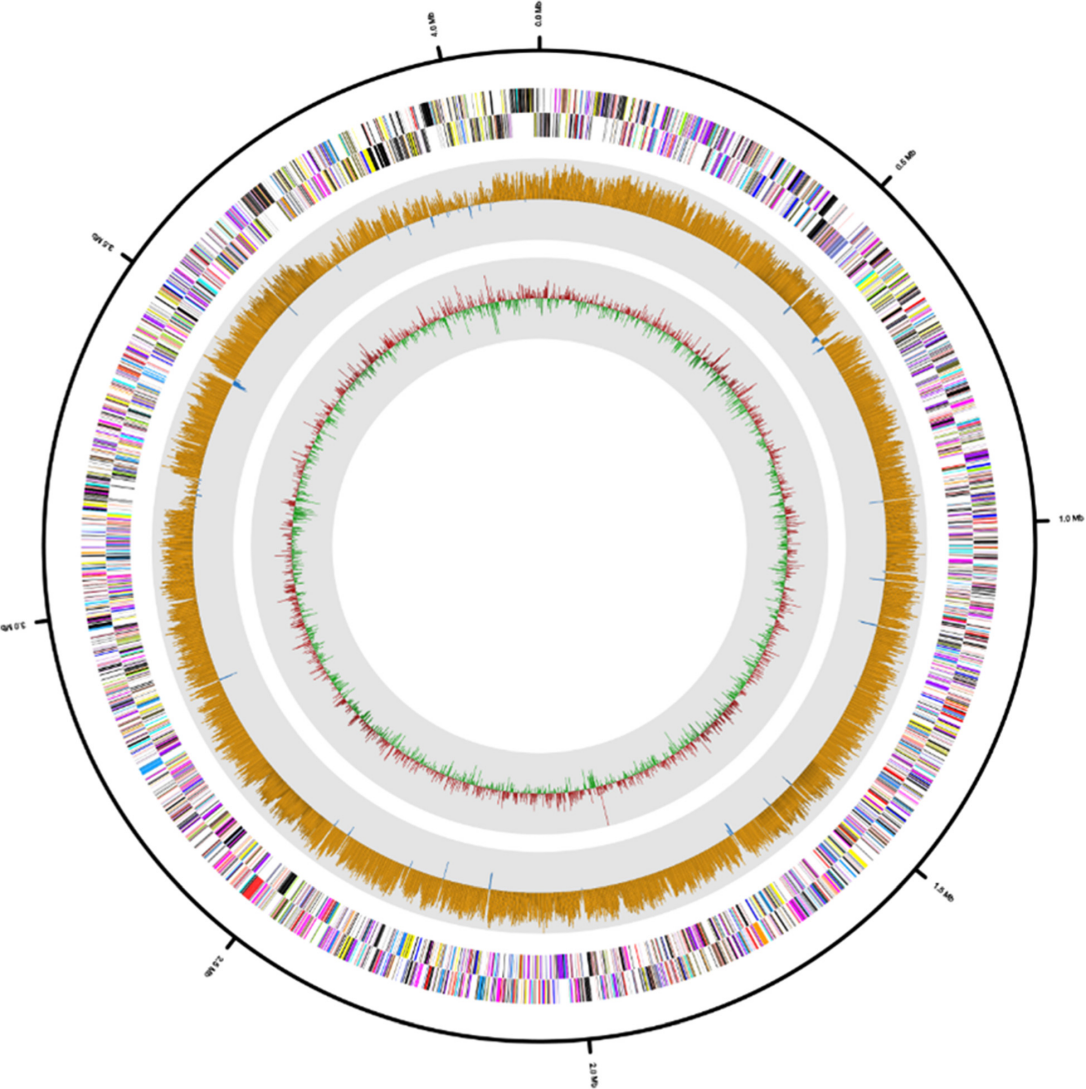
Graphical circular map of the *H. jeotgali* A29^T^ genome. RNA genes (red, tRNA and blue, rRNA) and genes on the reverse and forward strands (colored according to COG categories) are shown from the outside to the center. The inner circle shows the GC skew; yellow and blue indicate positive and negative values, respectively. GC content is indicated in red and green

The draft genome sequence of *H. jeotgali* A29^T^ was 4,131,621 bp and comprised three scaffolds including 20 contigs, and had a GC content of 64.9 % (Fig. 3 and Table 3). Of the 4,342 predicted genes, 4,215 were protein-coding and 2,636 ORFs (60.7 %) were assigned putative functions, whereas the remaining genes were annotated as hypothetical proteins. The genome contained 127 ORFs assigned to RNA genes, including 47 predicted for tRNA, 14 for rRNA (five 5S, two 16S, and seven 23S), and 66 for miscellaneous RNA (one archaeal signal recognition particle; five for the HgcC family; one archaeal RNA P; and 59 clustered regularly interspaced short palindromic direct repeat elements). The distribution of genes across COG functional categories is presented in Table 4.

**Table 3:** Genomic statistics

Attribute	Value	% of Total
Genome size (bp)	4,131,621	100.00
DNA coding (bp)	3,538,864	85.65
DNA G + C (bp)	2,682,192	64.92
DNA scaffolds	20	100.00
Total genes	4,342	100.00
Protein-coding genes	4,215	97.08
RNA genes	127	2.92
Genes in internal clusters	3,412	78.58
Genes with function prediction	2,636	60.71
Genes assigned to COGs	2,144	49.38
Genes with Pfam domains	2,638	60.76
Genes with signal peptides	79	1.82
Genes with transmembrane helices	984	22.66
CRISPR repeats	1

**Table 4:** Number of genes associated with general COG functional categories

Code	Value	% age	Description
J	154	6.53	Translation, ribosomal structure, and biogenesis
A	1	0.04	RNA processing and modification
K	107	4.54	Transcription
L	129	5.47	Replication, recombination, and repair
B	3	0.13	Chromatin structure dynamics
D	19	0.81	Cell cycle control, mitosis, and meiosis
Y	0	0.00	Nuclear structure
V	31	1.31	Defense mechanisms
T	78	3.31	Signal transduction mechanisms
M	69	2.93	Cell wall/membrane biogenesis
N	17	0.72	Cell motility
Z	0	0.00	Cytoskeleton
W	0	0.00	Extracellular structures
U	21	0.89	Intracellular trafficking, secretion, and vesicular transport
O	101	4.28	Posttranslational modification, protein turnover, chaperones
C	167	7.08	Energy production conversion
G	88	3.73	Carbohydrate transport metabolism
E	217	9.20	Amino acid transport metabolism
F	66	2.80	Nucleotide transport metabolism
H	131	5.56	Coenzyme transport metabolism
I	125	5.30	Lipid transport metabolism
P	158	6.70	Inorganic ion transport metabolism
Q	50	2.12	Secondary metabolites biosynthesis, transport catabolism
R	400	16.96	General function prediction only
S	226	9.58	Function unknown
-	2198	50.62	Not in COGs

The total is based on the total number of protein coding genes in the genome

## Conclusions

*H. jeotgali* A29^T^ encoded the genes associated with the mechanisms of salinity tolerance, biosynthesis and transport of compatible solutes such as glycine betaine (N,N,N-trimethylglycine) (choline sulfatase, choline dehydrogenase, betaine reductase, and glycine betaine transporter OpuD), ion exclusion using cation (Mg^2+^ and Cu^2+^) transport and K^+^ transport and Na^+^/H^+^ antiporter systems. The sequences may contribute to expansion of our knowledge of complex osmoregulation mechanism of the haloarchaea that should facilitate biotechnological applications of the haloarchaea and provide useful information on genetic mechanisms that enable haloarchaea to endure hypersaline environments.

## Abbreviations

ME: Minimum evolution
ML: Maximum likelihood
NJ: Neighbor-joining
PGM: Personal Genome Machine
